# Human MLH1 Protein Participates in Genomic Damage Checkpoint Signaling in Response to DNA Interstrand Crosslinks, while MSH2 Functions in DNA Repair

**DOI:** 10.1371/journal.pgen.1000189

**Published:** 2008-09-12

**Authors:** Qi Wu, Karen M. Vasquez

**Affiliations:** Department of Carcinogenesis, University of Texas M. D. Anderson Cancer Center, Science Park-Research Division, Smithville, Texas, United States of America; Stanford University School of Medicine, United States of America

## Abstract

DNA interstrand crosslinks (ICLs) are among the most toxic types of damage to a cell. For this reason, many ICL-inducing agents are effective therapeutic agents. For example, cisplatin and nitrogen mustards are used for treating cancer and psoralen plus UVA (PUVA) is useful for treating psoriasis. However, repair mechanisms for ICLs in the human genome are not clearly defined. Previously, we have shown that MSH2, the common subunit of the human MutSα and MutSβ mismatch recognition complexes, plays a role in the error-free repair of psoralen ICLs. We hypothesized that MLH1, the common subunit of human MutL complexes, is also involved in the cellular response to psoralen ICLs. Surprisingly, we instead found that MLH1-deficient human cells are more resistant to psoralen ICLs, in contrast to the sensitivity to these lesions displayed by MSH2-deficient cells. Apoptosis was not as efficiently induced by psoralen ICLs in MLH1-deficient cells as in MLH1-proficient cells as determined by caspase-3/7 activity and binding of annexin V. Strikingly, CHK2 phosphorylation was undetectable in MLH1-deficient cells, and phosphorylation of CHK1 was reduced after PUVA treatment, indicating that MLH1 is involved in signaling psoralen ICL-induced checkpoint activation. Psoralen ICLs can result in mutations near the crosslinked sites; however, MLH1 function was not required for the mutagenic repair of these lesions, and so its signaling function appears to have a role in maintaining genomic stability following exposure to ICL-induced DNA damage. Distinguishing the genetic status of MMR-deficient tumors as MSH2-deficient or MLH1-deficient is thus potentially important in predicting the efficacy of treatment with psoralen and perhaps with other ICL-inducing agents.

## Author Summary

Crosslinks, linking the complementary stands of the DNA double helix, can lead to cell death, because they are so effective at interfering with normal genomic transactions such as DNA replication. This property of crosslinking agents has long been utilized in cancer therapy. The purpose of our research is to understand the function of DNA repair proteins in cellular responses to DNA interstrand crosslinking agents. MSH2 is a central protein in the recognition of DNA mismatches, and we previously found that it plays an important role in protecting cells against the toxicity of crosslinks. The MLH1 protein functions in DNA mismatch repair in a later step, and we hypothesized that MLH1 may also be involved in repair of crosslinks. We were surprised to find that MLH1 function is important for DNA crosslink-induced signaling, rather than DNA repair. MLH1-deficient cells are more resistant to crosslinks and have defective signaling to processes that signal cell death. This work may have clinical consequences, as mutations in MSH2 and MLH1 are common in tumors. MSH2-deficient cells may be more vulnerable to DNA crosslink-inducing agents than normal, while MLH1-deficient cells have a greater potential to survive crosslinking treatment, which could instead potentiate further tumor initiation.

## Introduction

A DNA interstrand crosslink (ICL) is a type of DNA damage in which both strands of the DNA are covalently linked. ICLs present formidable challenges to the cell's essential DNA metabolic processes including replication and transcription. DNA crosslinking agents, e.g., psoralens, mitomycin C (MMC), platinum drugs, and nitrogen mustards, are among the most effective anticancer agents and are commonly included in combination chemotherapy regimens. The formation of covalent crosslinks is a critical event for the cytotoxicity and antitumor activity of these ICL-inducing agents [1]. Herbs that are rich in psoralens have been used for centuries to treat vitiligo and other skin disorders; and in modern medicine, psoralen is widely used in conjunction with ultraviolet A (UVA) irradiation [psoralen+UVA (PUVA)] for treatment of several skin disorders including psoriasis, mycosis fungoides, eczema, vitiligo and skin cancer [2]. UVA is ultraviolet light with a wavelength between 315–400 nm. While PUVA-induced ICLs have been extensively studied, the mechanism(s) of ICL processing in mammalian cells is still not well defined. Current evidence suggest that proteins from several DNA repair pathways are involved in the processing of ICLs in mammalian cells, including proteins with roles in nucleotide excision repair (NER), mismatch repair (MMR), and homologous recombination (HR) mechanisms [3]–[5]. As in bacteria and yeast, it is proposed that there is a major recombination-dependent error-free pathway and a minor recombination-independent error-generating pathway of ICL repair in human cells [3],[6]. However, the molecular details of these pathways are not yet clearly defined.

Triplex-forming oligonucleotides (TFOs) are single-stranded oligonucleotides that can bind to purine-rich stretches of duplex DNA via Hoogsteen hydrogen bonding in a sequence-specific manner. Psoralen-modified TFOs can induce site-specific psoralen ICLs at the duplex-triplex junction in both plasmid and genomic DNA [5], [7]–[9]. Studies employing this site-specific ICL model and others have demonstrated that NER proteins specifically recognize DNA-ICL lesions and are involved in an error-generating pathway of ICL repair [10]–[14], while the MMR protein MSH2 participates in the error-free repair of psoralen ICLs [3],[5],[6].

The DNA MMR system is essential for maintaining genomic stability and preventing tumor formation, and is highly conserved in evolution. MMR is responsible for correcting DNA replication errors and processing heteroduplex regions in HR intermediates [15]–[18]. In humans, the initial step of MMR is recognition of mismatches by one of two heterodimers, MutSα (MSH2 and MSH6) or MutSβ (MSH2 and MSH3). In the subsequent step, the mismatch bound by MutSα or MutSβ recruits a MutL complex, of which MLH1 is an essential component. It is thought that the MutS/MutL complex slides along the DNA until it encounters a strand break, and then loads exonuclease I to degrade the DNA strand containing the mispaired base. The resulting gap is then filled by Polymerase δ [19].

MMR recognition complexes can interact with several DNA lesions that are normally repaired by direct reversal, base excision repair, or NER; e.g. T-T cis-syn-cyclobutane pyrimidine dimers [20],[21], T-T 6-4 photoproducts [21], 8-oxoguanine [22],[23], O^6^-methylguanine [24],[25], O^4^-methylthymine [25], cisplatin intrastrand crosslinks [26],[27], and psoralen ICLs [4],[5].

MMR proteins have been shown to play a role in cell death in response to *N*-methyl-*N*′-nitro-*N*-nitrosoguanidine, 6-thioguanine, cisplatin, carboplatin, and B[a]P [28]–[31]. Recent studies show that MMR proteins are required for S-phase checkpoint activation induced by ionizing irradiation [32], and G2-checkpoint activation induced by cisplatin, S_N_1 DNA methylators, and 6-thioguanine [33]–[39]. Exposure to S_N_1 DNA methylators has been reported to activate MSH2- and MLH1-dependent phosphorylation of CHK1 through ATR [36],[37]. However, the function of MMR proteins in signaling cellular responses to psoralen ICLs has not been defined. Psoralen ICLs arrest human cells at S phase [40] by active checkpoint signaling [41]. Studies reveal that ATM and ATR may function as sensors in response to psoralen ICL exposure. The ICL-activated S-phase checkpoint depends on ATR-CHK1 and ATR-NBS1-FANCD2 pathways [42].

We have shown that MSH2, the common subunit of MutSα and MutSβ mismatch recognition complexes, plays a role in the error-free repair of psoralen ICLs [5]. Thus, we hypothesized that MLH1, the common subunit of human MutL complexes, is also involved in the cellular response to psoralen ICLs. Interestingly, we found that MLH1-deficient human cells are more resistant to psoralen ICLs, which is different from the MSH2-deficiency which results in sensitivity to this lesion [5]. We measured the apoptotic status of human MLH1-proficent and MLH1-deficient cells following induction of psoralen ICLs by caspase-3/7 activity and binding of annexin V, and found that in cells lacking MLH1 function, apoptosis was not as efficiently induced by psoralen ICLs as in MLH1-proficient cells. We also found that MLH1-deficient cells have reduced phosphorylation of CHK1 and CHK2 after induction of psoralen ICLs, suggesting that MLH1 is involved in signaling psoralen ICL-induced checkpoint activation. Importantly, MLH1 function is not required for the mutagenic repair of psoralen ICLs, suggesting it may have a role in maintaining genomic stability following exposure to ICL-induced DNA damage.

## Results

### MLH1-Deficient Human Cells Are Resistant to PUVA Treatment

**Figure 1:**
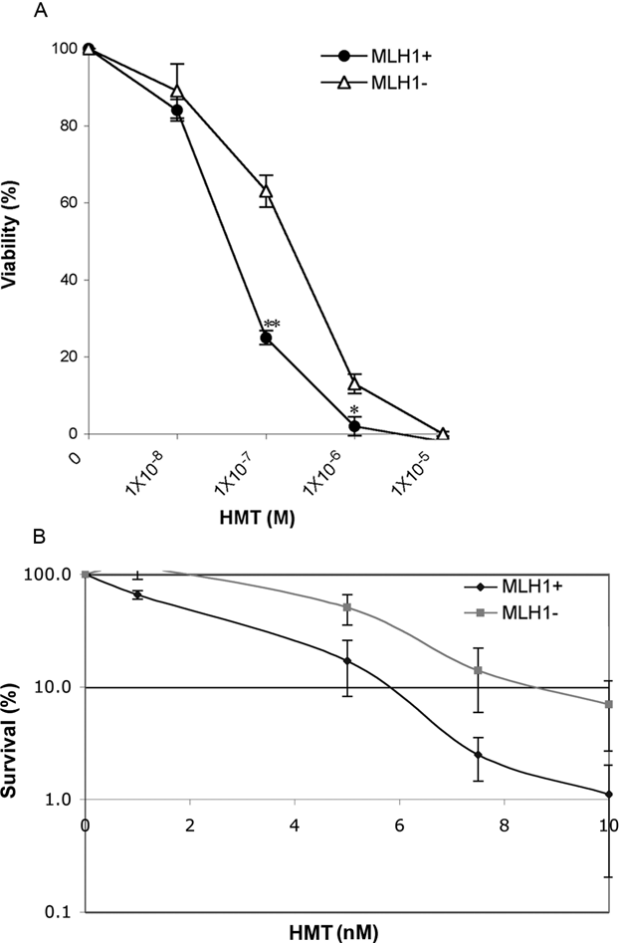
Sensitivity of MLH1-proficient or MLH1-deficient cells to PUVA treatment. (A) Viability curves are shown for MLH1+ (A2780) or MLH1− (A2780/cp70) cells treated with PUVA. The bars represent the standard errors of the means. **p<0.001, *p<0.01. (B) Results from clonogenic assays are shown for the same cells treated with PUVA.

To determine if MLH1 plays a role in processing psoralen-crosslinked DNA in human cells, we performed a cell viability assay following DNA damage induced by PUVA [cells were exposed to the psoralen derivative, 4′-hydroxymethyl-4,5′,8-trimethylpsoralen (HMT), at concentrations ranging from 10^−8^ to 10^−5^ M, and UVA irradiated at 1.8 J/cm^2^] in MLH1-proficient (A2780) and isogenic MLH1-deficient (A2780/cp70) ovarian cancer cells. Cell viability was measured 48 hours after PUVA treatment. The results shown in Figure 1A demonstrate that MLH1-deficient cells are ∼3-fold more resistant to PUVA treatment than MLH1-proficient cells at HMT concentrations between 10^−7^ and 10^−6^ M. Results from clonogenic assays also confirmed that MLH1-deficient A2780/cp cells showed a greater survivability after PUVA treatment than MLH1-proficient A2780 cells (Figure 1B).

To investigate whether this result was cell line specific, we used MLH1-specific siRNA oligonucleotides to reduce the level of MLH1 expression in human cervical cancer HeLa cells. Treatment of HeLa cells with 100 nM MLH1-specific siRNA oligonucleotides substantially reduced (∼70–90%) the level of MLH1 protein as assessed by western blotting (Figure S1A; see Text S1). Twenty-four hours after MLH1-specific or control siRNA treatment, HeLa cells were treated with HMT (from 10^−9^ to 10^−5^ M) and UVA irradiated at 1.8 J/cm^2^. Cell viability was measured 48 hours after treatment. Reduction in the level of MLH1 by siRNA oligonucleotide treatment rendered HeLa cells more resistant to psoralen ICLs when treated with 10^−7^ or 10^−6^ M HMT and UVA irradiation (Figure S2), consistent with the results obtained using the isogenic-paired A2780 cells under similar conditions.

### Apoptosis Is Not Efficiently Induced in MLH1-Deficient Cells in Response to PUVA Treatment

**Figure 2:**
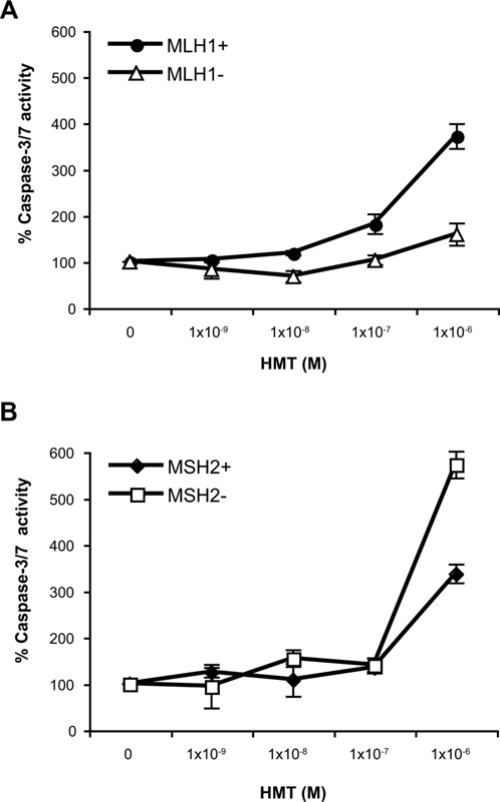
Caspase-3/7 activation in MLH1-deficient or MSH2-deficient human cells in response to PUVA treatment. (A) MLH1+ (A2780), MLH1− (A2780/cp70); (B) MSH2+ (HEC59+Chr2), MSH2− (HEC59) cells. The relative level of caspase-3/7 activity is shown for cells 48 hours after PUVA treatment. Caspase-3/7 activation was determined by cleavage of a caspase-3/7 substrate and performed in triplicate. The bars represent the standard errors of the means.

The difference in viability between MLH1-proficient and MLH1-deficient cells after exposure to PUVA led us to study the mechanism of cell death induced by this treatment. Apoptosis efficiency was determined by caspase-3/7 activity in PUVA treated MLH1-proficient and MLH1-deficient human cells 48 hours after treatment. As shown in Figure 2A, PUVA treatment induced apoptosis more effectively in the MLH1-proficient A2780 cells than in the MLH1-deficient cells. This finding suggests that MLH1 plays an important role in psoralen ICL-induced apoptosis. Unlike MLH1, MSH2 function is not required for psoralen ICL-induced apoptosis. Treatment with HMT (at 1×10^−6^ M) plus UVA irradiation at 1.8 J/cm^2^ induced apoptosis in both the MSH2-proficient HEC59+Chr2 cells and in the isogenic MSH2-deficient HEC59 cells 48 hours after treatment (Figure 2B).

**Figure 3:**
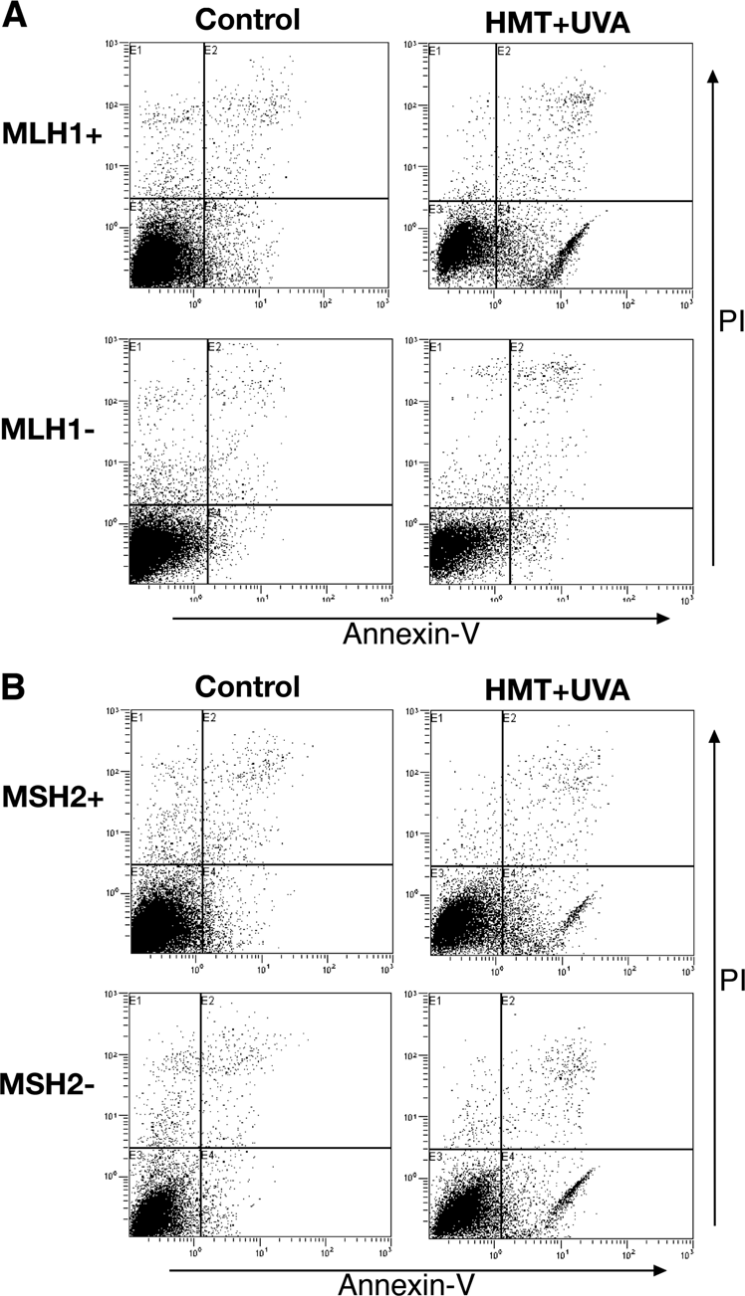
FACS Analysis of apoptotic cells after PUVA treatment in MLH1-deficient or MSH2-deficient human cells. (A) MLH1+ (A2780), MLH1− (A2780/cp70); (B) MSH2+ (HEC59+Chr2), and MSH2− (HEC59) cells were treated with 1×10^−6^ M HMT+UVA at 1.8 J/cm^2^. Forty-eight hours later, cells were first stained with annexin V-FITC and PI, then subjected to fluorescence-activated cell sorter analyses to identify apoptotic cells. The x-axis represents the staining level of annexin; the y-axis represents the staining level of PI. The lower right and upper right quadrants contain cells with annexin positive cells indicating the apoptotic cell population. The cell lines used in this study are indicated on the left of the figure and the treatment conditions are listed on top of the figure.

In order to determine the percentage of cells undergoing apoptosis after exposure to psoralen ICLs, flow cytometric-based annexin V-FITC–binding analyses were performed in MLH1-proficient and -deficient human cells treated with 1×10^−6^ M HMT and then irradiated with UVA at 1.8 J/cm^2^. The results shown in Figure 3A demonstrate that this treatment induced apoptosis in ∼62% of the A2780 cells 48 hours after induction of psoralen ICLs. In contrast, in the MLH1-deficient cells, only ∼5% of the treated cells had undergone apoptosis 48 hours after treatment. In the untreated control cells, ∼12% of the MLH1-proficient cells and ∼7% of the MLH1-deficient cells had undergone apoptosis. These data demonstrate that apoptosis is not efficiently induced in the A2780/cp70 MLH1-deficient cells after PUVA treatment. This result is consistent with the lack of caspase-3/7 activation in the A2780/cp70 cells treated in a similar fashion. We performed a similar assay in the MSH2-proficient and deficient human cells. As shown in Figure 3B, treatment with HMT (at 1×10^−6^ M)+UVA at 1.8 J/cm^2^, induced apoptosis in ∼15% of the HEC59+Chr2 cells and in ∼24% of the MSH2-deficient HEC59 cells 48 hours after induction of psoralen ICLs. In the untreated control cells, only approximately 5% of the MSH2-proficient or the MSH2-deficient cells had undergone apoptosis. These results confirmed that MLH1, but not MSH2 function is important for psoralen ICL-induced apoptosis.

### MLH1 Plays an Important Role in Signaling Psoralen ICL-Induced Checkpoint Activation

**Figure 4:**
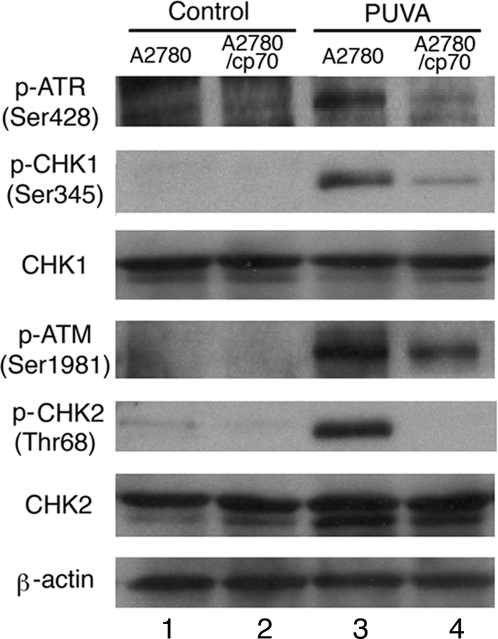
PUVA-induced checkpoint signaling in MLH1-proficient or MLH1-deficient cells. Lysates from MLH1-proficient (A2780) and MLH1-deficient (A2780/cp70) cells 1 hour following control (no treatment) or PUVA treatment (1×10^−6^ M HMT+1.8 J/cm^2^ UVA) were probed for phosphorylation of ATR (at Ser428), CHK1 (at Ser345), ATM (at Ser1981), CHK2 (at Thr68), total CHK1, total CHK2, and β-actin by western blotting. Lane 1: MLH1-proficient cells with no treatment; lane 2: MLH1-deficient cells with no treatment; lane 3: MLH1-proficient cells with PUVA treatment; lane 4: MLH1-deficient cells with PUVA treatment.

PUVA treatment can induce an S-phase cell cycle checkpoint in human cells [40]. The ICL-activated S-phase checkpoint depends on ATR-CHK1 and ATR-NBS1-FANCD2 pathways [42]. To determine if MLH1 function is involved in psoralen ICL-induced checkpoint signaling, we investigated the phosphorylation activation of ATR (assessed by phosphorylation at Ser428), CHK1 (at Ser345), ATM (at Ser1981), and CHK2 (at Thr68) in MLH1-proficient and MLH1-deficient human cells with or without PUVA treatment (1×10^−6^ M HMT+1.8 J/cm^2^). Phosphorylation of ATR (Ser428), CHK1 (Ser345), ATM (Ser1981) and CHK2 (Thr68) were observed at 1 hour following PUVA treatment in MLH1-proficient cells as shown in Figure 4. Interestingly, the phosphorylation level of ATR (Ser428) and CHK1 (Ser345) in MLH1-deficient cells was much lower than that detected in the MLH1-proficient cells (Figure 4). Strikingly, psoralen ICL-induced CHK2 phosphorylation was not detected in the MLH1-deficient cells, while similar levels of phosphorylation of ATM (Ser1981) were observed in both MLH1-proficient and MLH1-deficient cells (Figure 4). These results suggest that MLH1 participates in signaling ATR, CHK1, and CHK2 activation in response to psoralen ICLs in human cells.

### MLH1, Unlike MSH2, Is Not Directly Required for the Processing of Triplex-Directed Psoralen ICLs

We have reported previously that MSH2 is directly involved in the processing of psoralen ICLs [5]. To determine whether MLH1 protein function is required for the processing of psoralen ICLs, we subjected a psoralen crosslinked pSupFG1 plasmid to cell-free extracts either proficient or deficient in MLH1 function, together with [α-^32^P]dCTP, unlabelled dNTPs, and an ATP-regenerating system. Incorporation of radioactive dCTP into the vicinity of the ICL site indicates the occurrence of DNA repair synthesis. We found that a psoralen ICL induced similar levels of nucleotide incorporation into the 188 bp fragment containing the ICL site in both MLH1-proficient and MLH1-deficient cell extracts (Figure S3). This finding suggests that the ICL-induced repair synthesis does not depend on MLH1 function under the conditions of our assay.

### MLH1 Is Not Required for Psoralen ICL-Induced Mutagenesis

**Figure 5:**
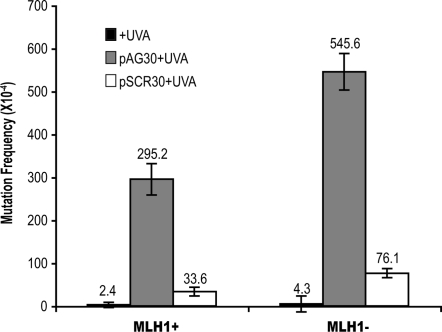
Psoralen ICL-induced mutagenesis in MLH1-proficient or MLH1-deficient human cell lines. MLH1-proficient (A2780) and MLH1-deficient cell lines (A2780/cp70) were transfected with the pSupFG1 mutation reporter plasmid and mutations in the *supF* reporter gene were measured 72 hours later. +UVA represents plasmid in the presence of UVA irradiation only at 1.8 J/cm^2^; pAG30+UVA represents pSupFG1 plasmid treated with the specific psoralen-modified TFO (pAG30) and then UVA irradiated at 1.8 J/cm^2^; and pSCR30 represents plasmid that was incubated with the psoralen-modified control oligonucleotide and UVA irradiated at 1.8 J/cm^2^. The bars represent the standard errors of the means of three independent experiments. The absolute mutation frequency is listed above each bar.

Psoralen ICLs can induce mutations in the DNA of both prokaryotic and eukaryotic cells [9], [43]–[46]. Since we have demonstrated that MLH1 deficiency reduces the loss of viability of cells exposed to psoralen ICLs, it is important to determine if MLH1 plays a role in the mutagenesis induced by these lesions. To examine whether MLH1 is involved in the error-generating repair of psoralen ICLs, we transfected psoralen-crosslinked pSupFG1 mutation reporter plasmids into MLH-proficient and -deficient human cells. Psoralen conjugated TFOs were used to direct a site-specific psoralen ICL into the *supF* mutation-reporter gene on the pSupFG1 plasmid. pAG30 is a psoralen conjugated TFO that binds specifically to *supF* gene sequences and can direct formation of ICLs at a specific site upon UVA irradiation. pSCR30 is a control TFO having the same base composition as pAG30, but in a scrambled sequence and so does not induce specific ICLs in the *supF* gene. Forty-eight hours after the cells were transfected with the ICL-damaged plasmids, DNA was isolated and digested with DpnI. DpnI is a restriction enzyme specific for methylated GATC sites, such that those plasmids that did not undergo replication in the mammalian cells will be digested by this enzyme and removed from further analysis. Next, plasmids were transfected into MB7070 cells, a *supF* mutation indicator strain of *E. coli*, to screen for *supF* gene mutations generated in the human cells. The background mutation frequency of the *supF* gene was 0.02% in MLH1-proficient ovarian cancer cells. As shown in Figure 5, the mutation frequency in the psoralen-crosslinked pSupFG1 plasmids recovered from MLH1-proficient cells was 2.9%, which is ∼120-fold greater than the background mutation frequency. In the MLH1-deficient cells, the background mutation frequency was 0.04%. When the psoralen-crosslinked pSupFG1 plasmids were processed in the MLH1-deficient cells, the mutation frequency was 5.6%, which is ∼130-fold greater than the background mutation frequency. In both MLH1-proficient and MLH1-deficient cells, psoralen ICLs can induce mutations more than 120-fold over background levels. These data suggest that MLH1 function is not required for the mutagenesis induced by psoralen ICLs in these cell lines.

The psoralen-crosslinked plasmids were also transfected into HeLa cells treated twice with MLH1-specific siRNA or control siRNA oligonucleotides, which resulted in undetectable levels of MLH1 as assessed by western blotting. MLH1 protein expression was reduced to below detectable levels during the 72 hour course of the assay (Figure S1B). The mutation frequencies of plasmids recovered from each treatment group are shown in Figure S4. In the untreated HeLa cells, the psoralen-ICL induced mutation frequency was 3.6%, which is ∼140-fold higher than the background mutation frequency of the untreated plasmid. In the control siRNA treated cells, the psoralen-ICL induced mutation frequency was ∼65-fold higher than the background level. The psoralen-crosslinked plasmids induced a mutation frequency ∼67-fold higher than the background frequency in the MLH1-specific siRNA treated cells, which is comparable to that (∼65-fold) in the control siRNA treated cells. Consistent with our results using the paired human ovarian cancer cells lines, these results suggest that loss of MLH1 does not diminish the mutagenic potential of triplex-directed psoralen ICLs in human cells, therefore MLH1 function is not required for the mutagenic processing of psoralen ICLs in HeLa cells.

### Psoralen ICL-Induced Mutations in MLH1-Proficient and -Deficient Human Cell Lines

Randomly selected clones containing psoralen-ICL induced mutations generated in the MLH1-proficient and MLH1-deficient ovarian cancer cells were sequenced and are listed in Figure S5. The mutants were selected from 3 different experiments. A total of 12 ICL-induced mutants obtained from the MLH1-proficient cells were sequenced. There was more than one mutation in the same colony in several cases. Ninety-two percent (11 of 12) of the mutations screened occurred in the predicated psoralen intercalation and crosslinking site (A_166_T_167_). Of these, 42% of the mutants (5 out of 12) consisted of T:A-A:T transversions at T_167_, 25% (3 of 12) contained A:T-T:A tranversions at A_166,_ 17% (2 out of 12) had T:A-G:C transversions at T_167_, one T:A-C:G transversion at T_167_ was identified, and one of the mutants consisted of a deletion containing the crosslinked site (Figure S5A).

As listed in Figure S5B, of the 14 mutants screened in the ICL-containing plasmids transfected into the MLH1-deficient cells, 86% (12 out of 14) contained mutations in the predicted psoralen crosslinking site. Of these, 29% (4 out 14) of the mutants contained T:A-A:T transversions at T_167_, 29% (4 of 14) contained A:T-T:A tranversions at A_166_, 14% (2 out of 14) had T:A-G:C transversions, 14% (2 out of 14) had single base deletions at T_167_, a single insertion at T_167_ was identified, and one mutant consisted of a deletion containing the crosslinked site. The mutation spectra generated in MLH1-proficient and MLH1-deficient human cells were very similar, suggesting that MLH1 is not required for this type of psoralen ICL-induced mutagenesis. Therefore, MLH1 function is not required in the error-generating processing of psoralen ICLs in these human cell lines.

## Discussion

The formation of DNA ICLs can lead to cell death by disrupting normal DNA functions such as replication and transcription. This cell-killing capacity of ICL-inducing agents has long been utilized in cancer therapy. However, ICL-inducing agents can also cause genomic instability, which may eventually lead to tumor formation. Genetic and biochemical studies have revealed the importance of proteins from several repair pathways in processing ICL lesions; these include proteins from the NER, MMR, and HR mechanisms. It is important to understand the functional significance of these proteins in both ICL-induced cellular responses and mutagenesis. MSH2, the common protein of MMR recognition complexes, plays important roles in both the cytotoxicity of psoralen ICLs and their error-free repair in human cells [4],[5],[47]. Since MLH1 functions downstream of MSH2 in MMR, we hypothesized that MLH1 may also be involved in these processes, which we have examined in this study.

### MSH2 and MLH1 Contribute Differently to Cytotoxicity of Psoralen ICL in Human Cells

We have previously demonstrated that MSH2 deficiency renders human cells more sensitive to psoralen ICLs [5]. Papouli et al. (2004) have reported that MLH1-proficient and -deficient human embryonic kidney cells show a similar level of sensitivity to PUVA treatment under their conditions (53). In their study they tested one concentration of psoralen (1 µM 4,5′,8-trimethyl-psoralen) and irradiated cells with 366 nm UVA at increasing doses (0–20 J/cm^2^). However, increasing the UVA dose may not correspond to an increased number of ICLs. Akkari et al. (2000) have demonstrated that increasing the concentrations of HMT used to treat cells does result in increased levels of ICLs (40). Therefore, in our study we varied the HMT concentrations (from 10^−8^ to 10^−5^ M), rather than the UVA dose (constant at 1.8 J/cm^2^). Our experimental results show that MLH1-deficient human ovarian and cervical cancer cells are more resistant to psoralen ICLs than isogenic MLH1-proficient cells. It is interesting that deficiencies in MSH2 versus MLH1 have different effects on cell survival in response to psoralen ICLs. This suggests that MSH2 and MLH1 have separate functions in response to DNA damage in addition to their traditional roles defined in MMR. We observed that MLH1, but not MSH2, is critical for psoralen ICL-induced apoptosis, which may account for the difference in cell survival between MSH2 and MLH1-deficient cells following PUVA treatment. Our results demonstrate that MLH1 is required for efficient activation of caspase 3/7, suggesting that MLH1 plays an important role in activating these apoptosis effector proteins.

We have shown that MSH2 is involved in the recognition and processing of psoralen ICLs in human cells [5]. Using triplex-directed psoralen ICL substrates and purified human recombinant proteins, we found that the human recombinant protein complex, MutSβ, can specifically bind to triplex-directed psoralen ICLs (data not shown), which is consistent with data reported by Zhang et al. (2002) [4], demonstrating that the human MutSβ complex can recognize psoralen ICLs. The interaction between the MSH2-MSH3 complex and MLH1 may mediate the MLH1-dependent apoptotic response to psoralen ICLs. Although we observed that psoralen ICLs result in increased apoptosis in MSH2-deficient cells and decreased apoptosis in MLH1-deficient cells (Figures 2 and 3), the increased apoptosis seen in MSH2-deficient cells may be due to failure to repair the ICL damage. ICLs present a formidable challenge to DNA metabolic activities, and may activate subsequent apoptotic pathways. Unlike our results with MSH2 [5], here we demonstrate that MLH1 function is not required for the processing of psoralen ICLs (Figure S4). Given that MSH2, but not MLH1 is involved in the recognition and processing of psoralen ICLs, it is likely that a non-canonical MMR function of MSH2, that circumvents a requirement for MLH1, is employed during the repair of psoralen ICLs. This is consistent with a previous report that processing of psoralen ICLs in mammalian cell extracts is dependent upon MutSβ, but is not dependent on the presence of MLH1 [4]. Therefore, MSH2, but not MLH1 is important for psoralen ICL repair in human cells. This provides a possible explanation for the differences in cell survival and apoptotic responses between MSH2- and MLH1-deficient cells following PUVA treatment.

### MLH1 Participates in Psoralen ICL-Induced Checkpoint Signaling

MLH1 has been reported to function in DNA damage-induced checkpoint signaling. For example, SN1 alkylating agents such as MNNG can activate MSH2- and MLH1-dependent phosphorylation of CHK1 through ATR [36]–[38], and MMR-dependent G2/M arrest by 6-TG signals through ATR-CHK1 [39]. In this study, we found that MLH1 is involved in psoralen ICL-induced ATR, CHK1, and CHK2 activation by phosphorylation. However, psoralen ICLs represent complex DNA lesions that differ from DNA damage induced by SN1 alkylating agents. For example, studies have shown that proteins from several repair pathways coordinately remove ICLs, including proteins from NER, MMR, and recombination mechanisms. Our results suggest that it is possible that the damage signal induced by psoralen ICLs can be passed to MLH1 in the absence of MSH2. Therefore, the cellular signaling in response to psoralen ICLs may differ from the signaling induced by SN1 alkylating agents. ATM has been shown to be required for phosphorylation of CHK2 at Thr68 in response to UV, ionizing radiation (IR), and replication blocks induced by hydroxyurea [48]. It is interesting that we observed that psoralen ICLs can activate ATM, but fail to activate CHK2 in MLH1-deficient cells. MSH2 and MLH1 have been shown to be required for CHK2 activation and S-phase checkpoint activation in IR-irradiated human cells [32]. Both *in vitro* and *in vivo* approaches demonstrate that MSH2 can bind to CHK2, and that MLH1 can associate with ATM [32]. The ATM activation and lack of CHK2 phosphorylation at Thr68 in psoralen-treated MLH1-deficient cells indicate that ATM requires MLH1, perhaps to interact with MSH2 and CHK2. ATR activity has been shown to be critical for a psoralen ICL-induced S-phase checkpoint [42]. We show here that MLH1 function is also important for psoralen ICL-induced checkpoint signaling. To our knowledge, this is the first demonstration that MLH1 is involved in the cellular response to psoralen ICLs in human cells.

### Clinical Relevance of MSH2 and MLH1 Status in Tumor Cells

Germline mutations in MSH2 and MLH1 together account for nearly half of all hereditary non-polyposis colorectal cancer (HNPCC) patients, of which ∼60% of the mutations are in the MLH1 gene, and ∼35% in the MSH2 gene [49]. Previously, we showed that MSH2 deficiency renders human cells more sensitive to psoralen ICLs and reduces the error-free repair of these lesions. Here we showed that MLH1 deficiency renders human cells more resistant to ICLs, likely by disruption of ICL-induced activation of apoptosis; and importantly, that MLH1 deficiency does not diminish the mutagenic repair of psoralen ICLs. Therefore, when treating tumors with ICL-inducing agents, the MSH2 and MLH1 status of the cells should be considered. For example, MSH2-deficient cells may be more vulnerable to ICL-inducing agents than MSH2-proficient cells, while MLH1-deficient cells have a greater potential to survive treatment with mutagenic ICL-inducing agents than MLH1-proficient cells, which may contribute to further tumor initiation.

## Materials and Methods

### Oligonucleotides and Mutation Reporter Plasmid, pSupFG1

Oligonucleotides, each containing an HMT moiety on the 5′ end and an amine group on the 3′ end, were synthesized by the Midland certified reagent company (Midland, TX). Both pSupFG1 and p2RT plasmids contain a *supF* mutation reporter gene, an ampicillin resistance gene, a pBR327 replication origin, and an SV40 viral replication origin.

### Cell Lines

A2780 (MLH1-proficient) and A2780/cp70 (MLH1-deficient) cells lines were provided by Dr. R. J. Legerski (University of Texas M.D. Anderson Cancer Center, Houston, TX) and were originally obtained from Dr. R. F. Ozols (Fox Chase Cancer Center, Pennsylvania, PA). Both cells lines were cultured in RPMI 1640 medium plus 10% fetal bovine serum (FBS). HeLa cells were maintained in Dulbecco's modified Eagle's medium with 10% FBS. The HEC59 (MSH2-deficient) cell line was cultured in DMEM/F12 medium plus 10% FBS. The HEC59+Chr2 (MSH2-proficient) cell line was maintained in DMEM/F12 medium containing 100 µg/ml G418 and 10% FBS.

### In Vitro Cytotoxicity Assays

The sensitivity of A2780 and A2780/cp70 cells to PUVA treatment was evaluated using an MTT assay (tetrazolium salt reduction, CellTiter 96 non-radioactive cell proliferation assay kit, Promega, Madison, WI). Briefly, 2×10^4^ cells were seeded in 96-well microplates in growth medium (100 µl) and incubated at 37°C in a humidified, 5% CO_2_ atmosphere. After 18 hours, the medium was removed and replaced with serum-free medium containing the corresponding concentrations of HMT (Sigma, St. Louis, MO) previously dissolved in DMSO and diluted in serum-free medium. After incubation in the dark for one hour, the cells were UVA irradiated for 30 minutes at 1 mW/cm^2^ to achieve a dose of 1.8 J/cm^2^. 15 W Cosmolux UVA lamps were used for irradiation and Mylar filters were used to filter out UVB and UVC irradiation (i.e. wavelengths <315 nm). Ice was placed near the cells and the temperature was maintained around 37°C during irradiation. The serum-free medium containing HMT was removed after irradiation and 100 µl growth medium was added to each well after washing the cells once using 100 µl serum-free medium. Triplicate cultures were established for each treatment. Forty-eight hours after UVA irradiation, cell viability was evaluated using an MTT assay. Viability was expressed as percentage of mean absorbance for treated wells compared to the mean absorbance for the control wells. Experiments were performed in triplicate for statistical analysis of variance (ANOVA) between MLH1-proficient and MLH1-deficient experimental groups. Clonogenic assays were carried out as described in Nairn *et al* [50]. PUVA treatment results in the production of both psoralen monoadducts and crosslinks. Psoralen monoadducts are efficiently processed by NER and since NER is functional in all cell lines tested in this study, we expect that the MMR status of the cells did not have a major effect in the response to PUVA-induced monoadducts. In support of this idea, published work by other groups suggest that loss of MMR has a minimal effect on UV-induced cytotoxicity in transformed and tumor-derived cell lines [51]–[53]. Since UV treatment results predominantly in intrastrand DNA adducts that are substrates for NER, we might expect a similar result with psoralen monoadducts.

### Western Blotting

Cells were lysed in ice-cold buffer containing 50 mM Tris HCL (pH 7.5), 1 mM EDTA, 10 mM DTT, 0.1% Triton X-100 and Complete™ proteinase inhibitor cocktail (Roche, Nutley, NJ). Cell lysates (50–100 µg) were mixed with SDS gel-loading buffer and heated at 95°C for 10 min, separated electrophoretically on a 7.5% or 10% SDS-polyacrylamide gel, and transferred to polyvinylidene difluoride membranes (Bio-Rad Laboratories. Inc, Hercules, CA). The blots were blocked for 1 hour in tris buffered saline (TBS) containing 5% nonfat milk and 0.1% Tween 20. The blots were then incubated with diluted primary antibody overnight at 4°C. Primary antibodies used in this study include rabbit anti-human MLH1 (Calbiochem, San Diego, CA), mouse anti-human p-ATM (Ser1981), rabbit anti-human p-ATR (Ser428), p-CHK1 (Ser345), CHK1, p-CHK2 (Thr68), CHK2 (Santa Cruz Biotechnology, Santa Cruz, CA), mouse-anti human β-actin, rabbit anti-human PCNA antibody, and rabbit anti-human GAPDH polyclonal antibody (Santa Cruz Biotechnology, Santa Cruz, CA). The blots were washed three times with TBS containing 0.1% Tween 20 and incubated for 1 hour with horseradish peroxidase-conjugated goat anti rabbit IgG or mouse IgG secondary antibodies (Bio-Rad, Hercules, CA). After three washes with TBS containing 0.1% Tween 20, bound secondary antibody was detected by using an ECL detection reagent (Amersham, Milano, Italy).

### Caspase 3/7 Cleavage Assay

2×10^4^ cells were seeded in 96-well microplates in growth medium (100 µl) and incubated at 37°C in a humidified, 5% CO_2_ atmosphere. Eighteen hours later, psoralen ICLs were induced in the cells by PUVA treatment as described above. Triplicate cultures were established for each treatment. Forty-eight hours after UVA irradiation, the apoptotic status was evaluated by activation of caspase-3/7 using the Apo-ONE homogeneous caspase-3/7 assay (Promega, Madison, WI). The activation of caspase-3/7 is indicated by cleavage of a caspase-3/7 substrate that can be measured by fluorescence. The level of apoptosis was expressed as a percentage of mean fluorescence for each PUVA treated sample compared to the mean fluorescence for the control sample.

### Annexin V-FITC–Binding Assay and Fluorescence-Activated Cell Sorter (FACS) Analysis

Annexin V-FITC apoptosis detection kit I (BD Biosciences Pharmingen, San Diego, CA) was used to quantitatively measure apoptotic cells. Forty-eight hours after treatment with 1×10^−6^ M HMT plus 1.8 J/cm^2^ UVA irradiation, both floating and attached cells were harvested. The cells were washed twice with ice cold PBS and then resuspended in 1× binding buffer [10 mM Hepes/NaOH (pH 7.4) 140 mM NaCl, 2.5 mM CaCl_2_] at 1×10^6^ cells/ml. 200 µl of the solution (2×10^5^ cells) was transferred to a 5 ml culture tube. Cells were gently vortexed and incubated with 10 µl of annexin V-FITC and 10 µl of propidium iodide (PI) for 30 min at room temperature (25°C) in the dark. 280 µl of 1× binding buffer was added to each tube. Samples were analyzed on Beckman-Coulter Ultra flow cytometer within one hour and analyzed with Expo32 software. The instrument was set up with an argon (488 nm) laser for excitation and a 525 nm pass filter for the FITC label and a 630 nm pass filter for PI with appropriate compensation. Annexin V-FITC is used to quantitatively determine the percentage of cells that are undergoing apoptosis, which relies on the fact that cells lose membrane asymmetry in the early phases of apoptosis. PI is a standard viability probe and is used to distinguish viable from nonviable cells in FACS analysis. Viable cells with intact membranes exclude PI, whereas the membranes of dead and damaged cells are permeable to PI. Cells that stain positive for annexin V-FITC and negative for PI are undergoing apoptosis. Cells that stain positive for both annexin V-FITC and PI are either in the end stage of apoptosis, are undergoing necrosis, or are already dead. Cells that stain negative for both annexin V-FITC and PI are alive and not undergoing measurable apoptosis.

### Mutagenesis Assays

Psoralen crosslinked pSupFG1 plasmid was transfected into human cells using Gene-PORTER transfection reagent (Gene Therapy System, Inc. San Diego, CA). Approximately 5 µg of plasmid DNA was used per 5×10^5^ human cells. The cells were incubated 48 hours prior to the isolation of the plasmid DNA. The plasmid was subjected to DpnI restriction enzyme digestion to remove unreplicated DNA, followed by phenol-chloroform extraction, and transformation into *E. coli* MBM7070 indicator strain, which carries an amber mutation in the *LacZ* gene. Mutations in the *supF* gene can be detected using a blue/white screen on 5-bromo-4-chloro-3-indolyl-Β-D-galactoside, isopropyl β-D-thiogalactoside, and ampicillin plates. The mutation frequency was determined as the number of mutant colonies (white colonies) to the total number of colonies (blue+white colonies). Experiments were performed in triplicate. DNA was isolated from randomly selected colonies for DNA sequencing analysis.

## Supporting Information

Figure S1 Downregulation of MLH1 expression by siRNA treatment in HeLa cells. (A) The relative levels of MLH1 protein are shown for cells treated with PBS, 100 nM control siRNA, or 100 nM MLH1-specific siRNA oligonucleotide at 24 hours, 48 hours and 72 hours after treatment. The bars represent the standard errors of the means of protein expression assessed by western blotting from four independent experiments. MLH1-specific siRNA reduced MLH1 protein expression to ∼29% compared to a control non-targeting siRNA after 24 hours. Forty-eight hours and 72 hours after the MLH1-specific siRNA transfection, the remaining MLH1 level was ∼13% and ∼21% of control, respectively. (B) Western blot showing the levels of MLH1 protein following treatment with MLH1-specific siRNA oligonucleotide on days 1, 2, and 3 during the mutagenesis assay. GAPDH and PCNA protein levels were used as loading controls for cytoplasm and nuclear proteins, respectively. HeLa cells were transfected twice with 100 nM siRNA oligonucleotides on day −3 and day 0.
(2.92 MB TIF)

Figure S2 Sensitivity of MLH1 specific siRNA or control siRNA oligonucleotide treated HeLa cells to PUVA treatment. Cell viability was determined 48 hours after PUVA treatment using an MTT assay performed in triplicate. The bars represent the standard error of the means. **p<0.001, *p<0.01.
(8.91 MB TIF)

Figure S3 Nucleotide incorporation into plasmid, p2RT, in the vicinity of TFO-targeted psoralen ICLs in MLH1-proficient or MLH1-deficient human cell extracts. (A) Ethidium-bromide stained gel; and (B) autoradiogram showing DNA synthesis stimulated by ICL formation in the p2RT plasmid, as measured by incorporation of radiolabeled nucleotides into a 190 bp restricted fragment containing the psoralen ICL site. Lane 1: p2RT plasmid incubated with MLH1-proficient cell extract; lane 2: p2RT containing a triplex-targeted ICL incubated with MLH1-proficient cell extract; lane 3: p2RT plasmid incubated with MLH1-deficient cell extract; lane 4: p2RT containing a triplex-targeted ICL incubated with MLH1-deficient cell extract.
(2.00 MB TIF)

Figure S4 Psoralen ICL-induced mutagenesis in siRNA treated HeLa cells. The mutation frequency of the *supF* gene was determined as the ratio of the number of mutant colonies (white colonies) to the total colonies (blue+white colonies). −UVA represents pSupFG1 without treatment; +UVA represents plasmid in the presence of UVA irradiation at 1.8 J/cm^2^; pAG30+UVA represents pSupFG1 plasmid treated with the specific psoralen-modified TFO (pAG30) at 10^−6^ M and then UVA irradiated at 1.8 J/cm^2^; and pSCR30 represents plasmid that was incubated with the psoralen-modified control oligonucleotide (10^−6^ M) and UVA irradiated (1.8 J/cm^2^). The bars represent the standard errors of the means of three independent experiments.
(16.63 MB TIF)

Figure S5 Psoralen-induced mutations in MLH1-proficient or MLH1-deficient human ovarian cancer cell lines. Mutation spectra of the psoralen ICL-induced mutations in the *supF* gene in the (A) MLH1-proficient A2780 cell line, and (B) MLH1-deficient A2780/cp70 cell line. Base substitutions are listed above the *supF* gene sequence. Base deletions are indicated by a ‘−’. Base insertions are indicated by a ‘+’. Multiple mutations in the same plasmid are underlined and listed in the same line. The TFO-binding site is underlined. The targeted TA site for psoralen ICL formation is indicated by boldface type.
(16.79 MB TIF)

Text S1 Supplemental text.
(0.06 MB DOC)
