# Supplementary figures and images for: Human MLH1 Protein Participates in Genomic Damage Checkpoint Signaling in Response to DNA Interstrand Crosslinks, while MSH2 Functions in DNA Repair
Source: PLoS Genet. 2008 Sep 12;4(9):e1000189. doi: 10.1371/journal.pgen.1000189 (PMC2526179; doi:10.1371/journal.pgen.1000189)

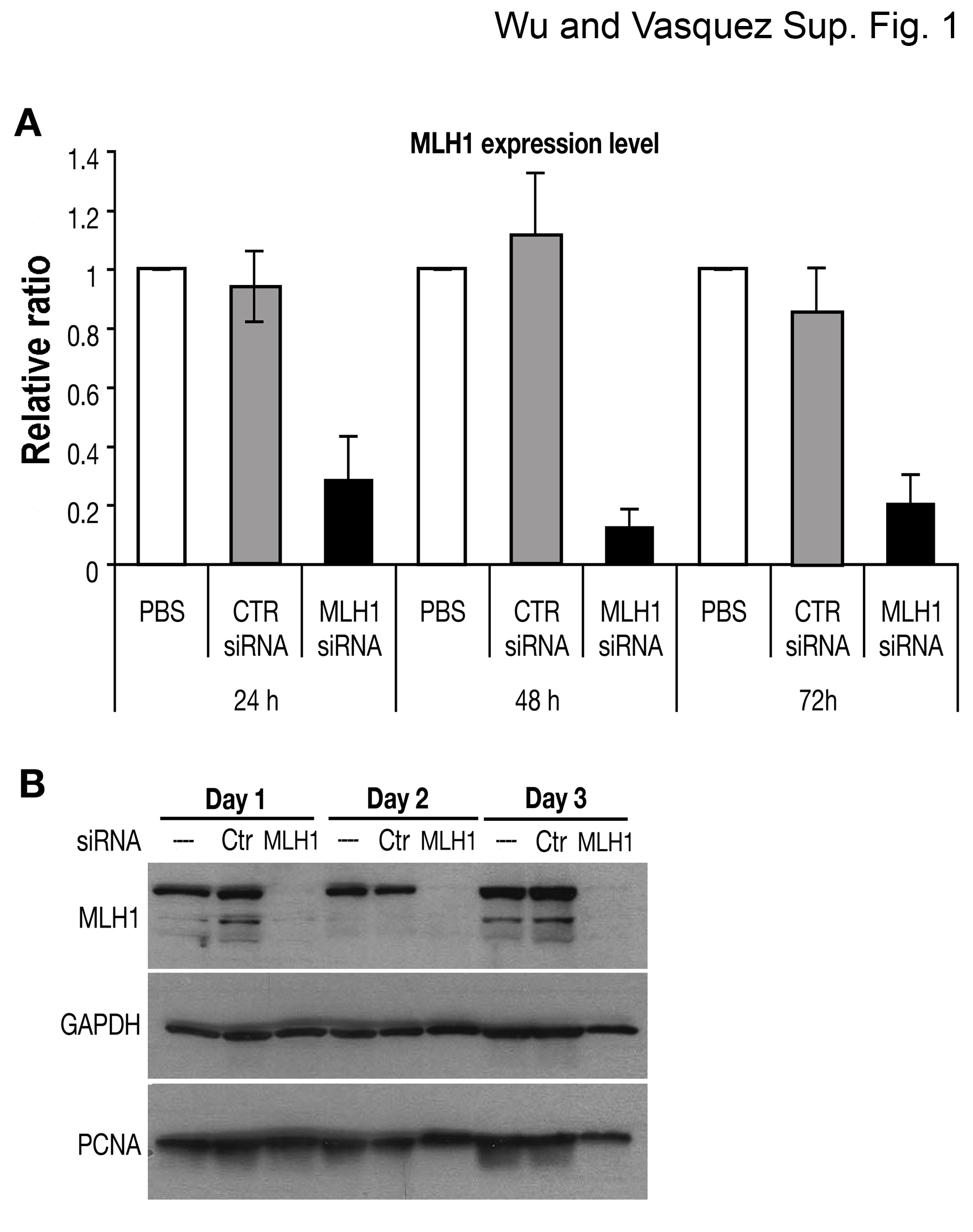

Supplement: Figure S1 — Downregulation of MLH1 expression by siRNA treatment in HeLa cells. (A) The relative levels of MLH1 protein are shown for cells treated with PBS, 100 nM control siRNA, or 100 nM MLH1-specific siRNA oligonucleotide at 24 hours, 48 hours and 72 hours after treatment. The bars represent the standard errors of the means of protein expression assessed by western blotting from four independent experiments. MLH1-specific siRNA reduced MLH1 protein expression to ∼29% compared to a control non-targeting siRNA after 24 hours. Forty-eight hours and 72 hours after the MLH1-specific siRNA transfection, the remaining MLH1 level was ∼13% and ∼21% of control, respectively. (B) Western blot showing the levels of MLH1 protein following treatment with MLH1-specific siRNA oligonucleotide on days 1, 2, and 3 during the mutagenesis assay. GAPDH and PCNA protein levels were used as loading controls for cytoplasm and nuclear proteins, respectively. HeLa cells were transfected twice with 100 nM siRNA oligonucleotides on day −3 and day 0. (2.92 MB TIF) [file pgen.1000189.s001.tif]

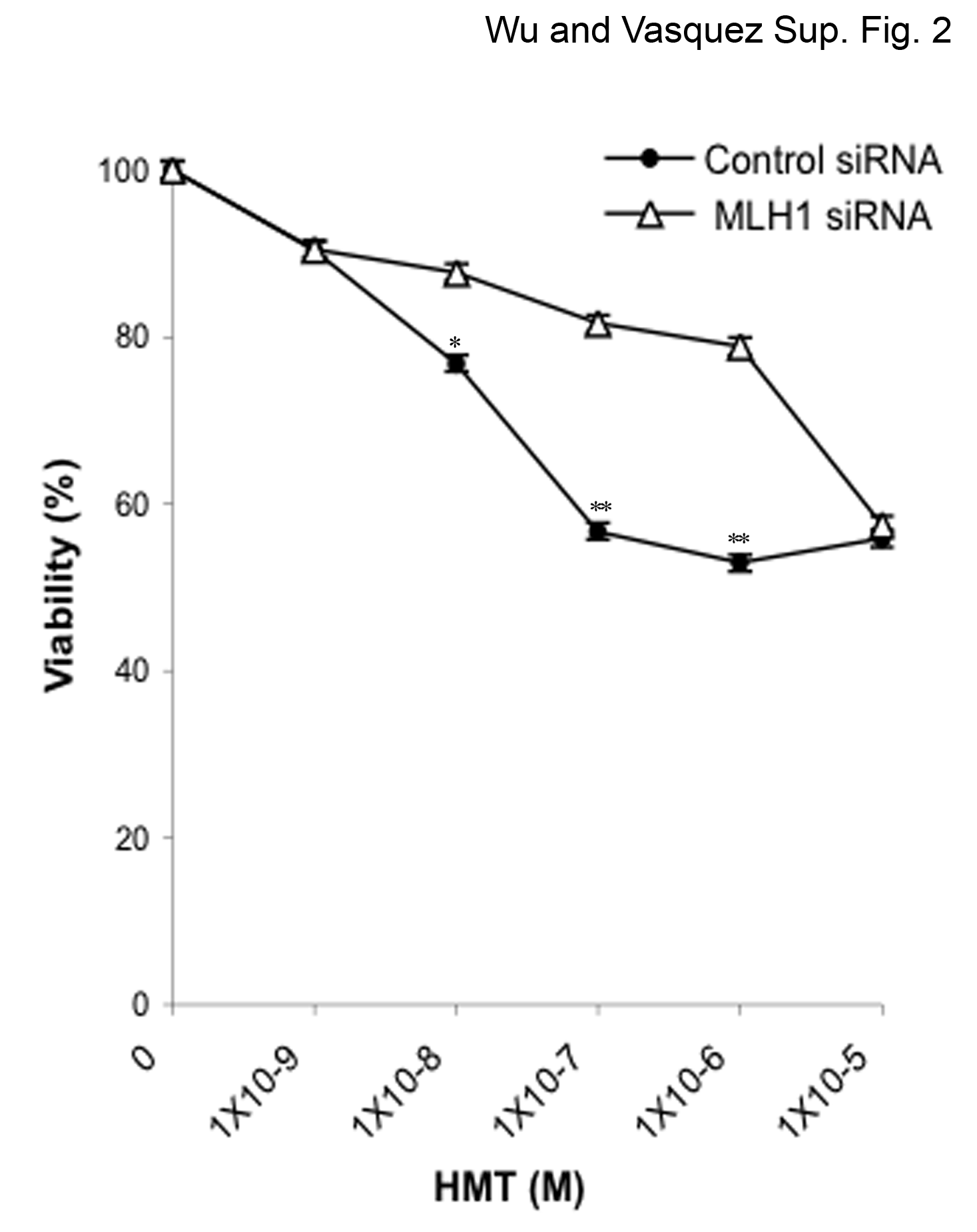

Supplement: Figure S2 — Sensitivity of MLH1 specific siRNA or control siRNA oligonucleotide treated HeLa cells to PUVA treatment. Cell viability was determined 48 hours after PUVA treatment using an MTT assay performed in triplicate. The bars represent the standard error of the means. **p<0.001, *p<0.01. (8.91 MB TIF) [file pgen.1000189.s002.tif]

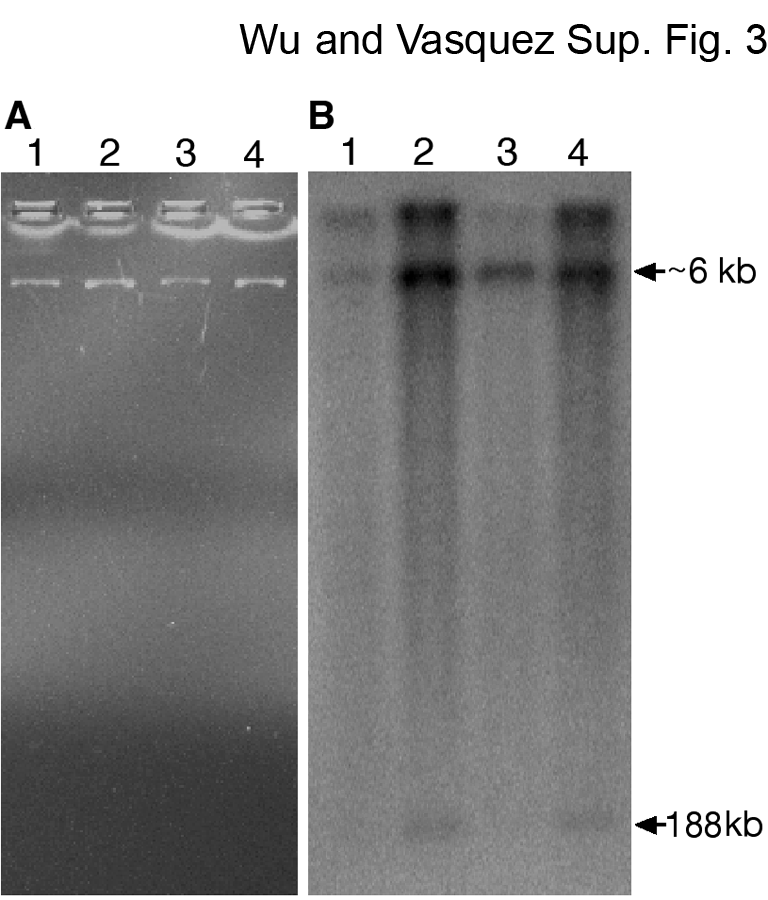

Supplement: Figure S3 — Nucleotide incorporation into plasmid, p2RT, in the vicinity of TFO-targeted psoralen ICLs in MLH1-proficient or MLH1-deficient human cell extracts. (A) Ethidium-bromide stained gel; and (B) autoradiogram showing DNA synthesis stimulated by ICL formation in the p2RT plasmid, as measured by incorporation of radiolabeled nucleotides into a 190 bp restricted fragment containing the psoralen ICL site. Lane 1: p2RT plasmid incubated with MLH1-proficient cell extract; lane 2: p2RT containing a triplex-targeted ICL incubated with MLH1-proficient cell extract; lane 3: p2RT plasmid incubated with MLH1-deficient cell extract; lane 4: p2RT containing a triplex-targeted ICL incubated with MLH1-deficient cell extract. (2.00 MB TIF) [file pgen.1000189.s003.tif]

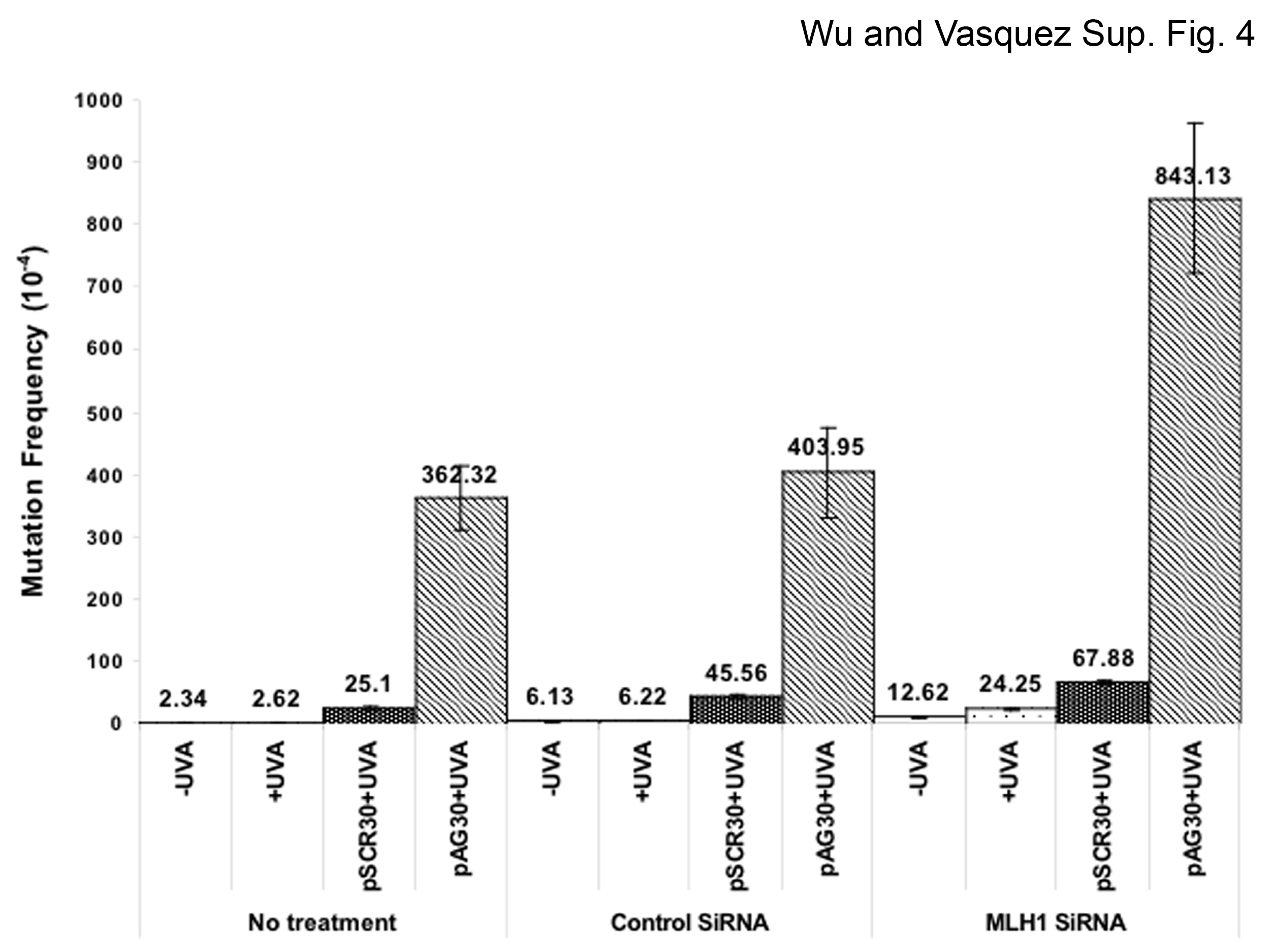

Supplement: Figure S4 — Psoralen ICL-induced mutagenesis in siRNA treated HeLa cells. The mutation frequency of the supF gene was determined as the ratio of the number of mutant colonies (white colonies) to the total colonies (blue+white colonies). −UVA represents pSupFG1 without treatment; +UVA represents plasmid in the presence of UVA irradiation at 1.8 J/cm2; pAG30+UVA represents pSupFG1 plasmid treated with the specific psoralen-modified TFO (pAG30) at 10−6 M and then UVA irradiated at 1.8 J/cm2; and pSCR30 represents plasmid that was incubated with the psoralen-modified control oligonucleotide (10−6 M) and UVA irradiated (1.8 J/cm2). The bars represent the standard errors of the means of three independent experiments. (16.63 MB TIF) [file pgen.1000189.s004.tif]

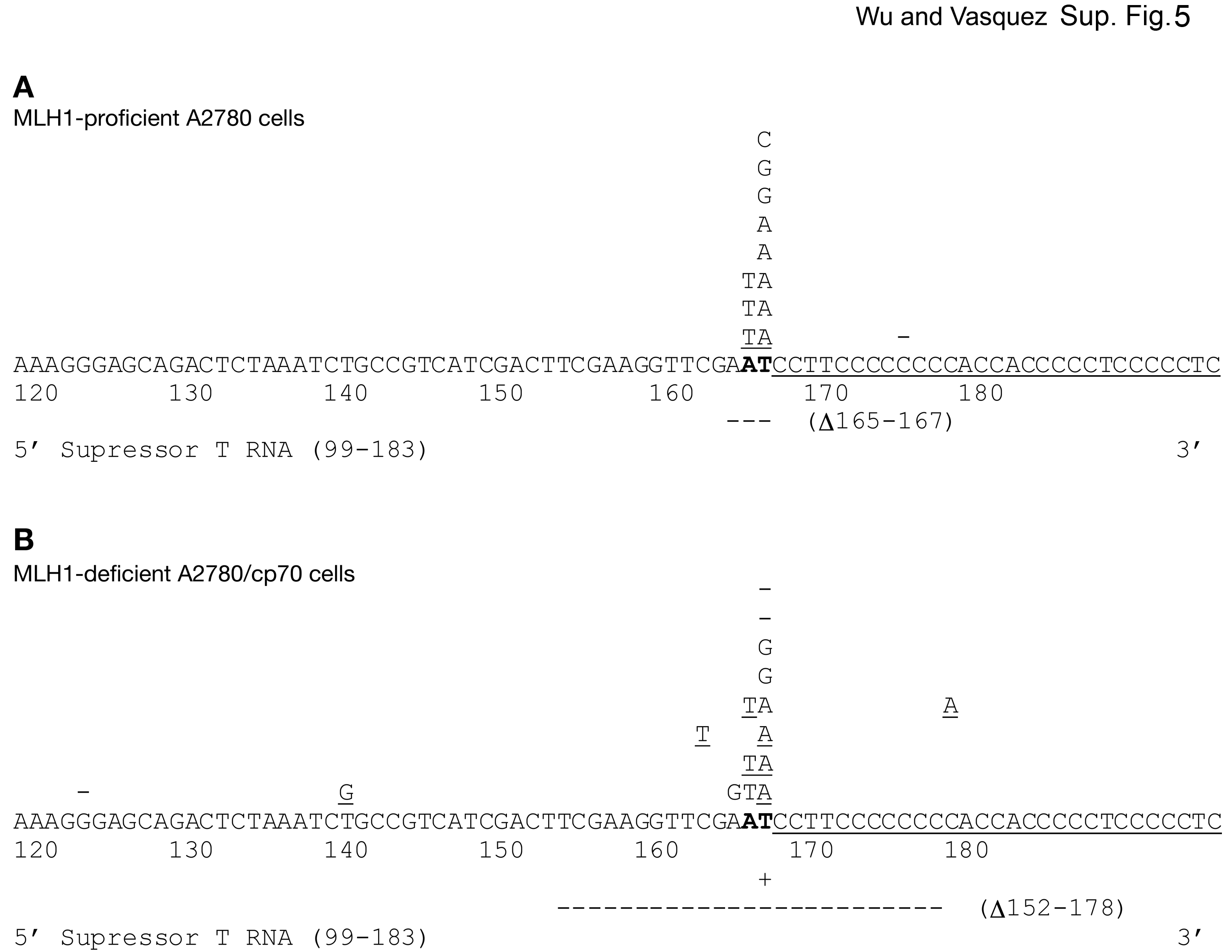

Supplement: Figure S5 — Psoralen-induced mutations in MLH1-proficient or MLH1-deficient human ovarian cancer cell lines. Mutation spectra of the psoralen ICL-induced mutations in the supF gene in the (A) MLH1-proficient A2780 cell line, and (B) MLH1-deficient A2780/cp70 cell line. Base substitutions are listed above the supF gene sequence. Base deletions are indicated by a ‘−’. Base insertions are indicated by a ‘+’. Multiple mutations in the same plasmid are underlined and listed in the same line. The TFO-binding site is underlined. The targeted TA site for psoralen ICL formation is indicated by boldface type. (16.79 MB TIF) [file pgen.1000189.s005.tif]
