# Supplementary material for: Human MLH1 Protein Participates in Genomic Damage Checkpoint Signaling in Response to DNA Interstrand Crosslinks, while MSH2 Functions in DNA Repair
Source: PLoS Genet. 2008 Sep 12;4(9):e1000189. doi: 10.1371/journal.pgen.1000189 (PMC2526179; doi:10.1371/journal.pgen.1000189)
Supplement: Text S1 — Supplemental text. (0.06 MB DOC) [file pgen.1000189.s006.doc]

**Supplemental Methods and Figures**

**siRNA treatment.** The sequence of the siRNA oligonucleotide designed to target human MLH1 was: C.A.G.U.G.U.A.U.G.C.A.G.C.C.U.A.U.U.U. The 2′-deprotected and desalted siRNA duplex was synthesized by Dharmacon, Inc. (Chicago, IL). Control siRNA, siCONTROL non-targeting siRNA #1, was purchased from Dharmacon, Inc. The level of MLH1 protein reduced by specific siRNA treatment was evaluated by western analysis (described below) as shown in Supplemental Figure 1.

***In vitro* cytotoxicity assay using HeLa cells treated with siRNA.** 200,000 HeLa cells were plated in a 10 cm dish on day -2. On day -1 the cells we transfected with DarmaFect^TM^ 1 (Dharmacon, Inc., Chicago, IL) transfection reagent and siRNA at a total concentration of 100 nM. Eight hours after siRNA treatment, 2x10^4^ cells were seeded in 96-well microplates in growth medium (100 µl) and incubated at 37ºC in a humidified, 5% CO_2_ atmosphere. On day 0, the medium was removed and replaced with serum-free medium containing the noted concentrations of HMT. After 1 hour of incubation in dark, the cells were irradiated as described above. The serum-free medium containing HMT was removed after UVA irradiation and 100 µl growth medium was added to each well after washing the cells once using 100 µl serum-free medium. Triplicate cultures were established for each treatment. On day 5, the sensitivity of MLH1 siRNA and control siRNA treated cells to PUVA treatment was measured using an MTT cell viability assay using similar procedures as for the A2780 cells described above. The MLH1 protein level in either MLH1-specific or control siRNA treated HeLa cells was assayed by western blotting on days 1 and 2. Experiments were performed in triplicate for statistical analysis (ANOVA) between experimental groups.

***In vitro* nucleotide incorporation assays.** A2780 and A2780/cp70 cell extracts were prepared from cells pellets using NucBuster^TM^ protein extraction kit (Novagen, Madison, WI). The final salt concentration was 200 mM. Supercoiled p2RT plasmid (~6 kb), was coincubated with psoralen-modified TFOs (1x10^-6^ M) and UVA irradiated (at 1.8 J/cm^2^) to induce site-specific psoralen ICLs in the *supF* mutation reporter gene. The DNA (300 ng) was then added to whole cell extracts (100 μg protein) supplemented with 2 μCi [α-^32^P]dCTP and 20 μM each of dATP, dGTP, and dTTP in a repair buffer containing (45 mM HEPES-KOH, pH 7.7, 70 mM KCl, 7.4 mM MgCl_2_, 0.9 mM DTT, 0.4 mM EDTA, 2 mM ATP, 40 mM phosphocreatine, 2.5 μM creatine phosphokinase, 3.4% glycerol, and 18 μg BSA) as described previously (Wu et al., 2005). The reactions were incubated at 30ºC for 3 hours and then extracted with phenol-chloroform, double digested with EcoRI and SacI restriction enzymes to release a 190 bp fragment containing the ICL site, and analyzed by agarose gel electrophoresis. Visualization of plasmid DNA and the incorporated [α-^32^P]-dCTP was achieved by ethidium bromide staining and autoradiography, respectively.

**Mutagenesis assay using the siRNA-treated HeLa cells.** 100,000 HeLa cells were plated in a 10 cm dish on day -4. On day -3 the cells were transfected with either PBS, MLH1-specific siRNA, or control non-targeting siRNA at a total concentration of 100 nM. On day 0, the cells were again transfected with either PBS, MLH1-specific siRNA, or control siRNA and incubated in growth media at 37ºC in a humidified, 5% CO_2_ atmosphere. On day 1, psoralen crosslinked pSupFG1 plasmid was transfected into PBS treated or siRNA treated HeLa cells using Gene-PORTER transfection reagent (Gene Therapy System, Inc. San Diego, CA). Approximately 5 μg of plasmid DNA was used per 5x10^5^ cells. The cells were incubated in growth medium at 37ºC in a humidified, 5% CO_2_ atmosphere for 48 hours. On day 3 the plasmid DNA was isolated and subjected to DpnI restriction enzyme digestion to remove unreplicated DNA, followed by phenol-chloroform extraction, and transformation into *E. coli* MBM7070 indicator strain to detect mutations. The mutation frequency of the *supF* gene was determined as the ratio of the number of mutant colonies (white colonies) to the total colonies (blue+white colonies). Experiments were performed in triplicate.
